# Evaluating the Impact of Adaptive Personalized Goal Setting on Engagement Levels of Government Staff With a Gamified mHealth Tool: Results From a 2-Month Randomized Controlled Trial

**DOI:** 10.2196/28801

**Published:** 2022-03-31

**Authors:** Raoul Nuijten, Pieter Van Gorp, Alireza Khanshan, Pascale Le Blanc, Pauline van den Berg, Astrid Kemperman, Monique Simons

**Affiliations:** 1 Department of Industrial Engineering Eindhoven University of Technology Eindhoven Netherlands; 2 Department of Industrial Design Eindhoven University of Technology Eindhoven Netherlands; 3 Department of the Built Environment Eindhoven University of Technology Eindhoven Netherlands; 4 Department of Social Sciences Wageningen University and Research Wageningen Netherlands

**Keywords:** mHealth, health promotion, physical activity, personalization, adaptive goal setting, gamification, office workers

## Abstract

**Background:**

Although the health benefits of physical activity are well established, it remains challenging for people to adopt a more active lifestyle. Mobile health (mHealth) interventions can be effective tools to promote physical activity and reduce sedentary behavior. Promising results have been obtained by using gamification techniques as behavior change strategies, especially when they were tailored toward an individual’s preferences and goals; yet, it remains unclear how goals could be personalized to effectively promote health behaviors.

**Objective:**

In this study, we aim to evaluate the impact of personalized goal setting in the context of gamified mHealth interventions. We hypothesize that interventions suggesting health goals that are tailored based on end users’ (self-reported) current and desired capabilities will be more engaging than interventions with generic goals.

**Methods:**

The study was designed as a 2-arm randomized intervention trial. Participants were recruited among staff members of 7 governmental organizations. They participated in an 8-week digital health promotion campaign that was especially designed to promote walks, bike rides, and sports sessions. Using an mHealth app, participants could track their performance on two social leaderboards: a leaderboard displaying the individual scores of participants and a leaderboard displaying the average scores per organizational department. The mHealth app also provided a news feed that showed when other participants had scored points. Points could be collected by performing any of the 6 assigned tasks (eg, walk for at least 2000 m). The level of complexity of 3 of these 6 tasks was updated every 2 weeks by changing either the suggested task intensity or the suggested frequency of the task. The 2 intervention arms—with participants randomly assigned—consisted of a personalized treatment that tailored the complexity parameters based on participants’ self-reported capabilities and goals and a control treatment where the complexity parameters were set generically based on national guidelines. Measures were collected from the mHealth app as well as from intake and posttest surveys and analyzed using hierarchical linear models.

**Results:**

The results indicated that engagement with the program inevitably dropped over time. However, engagement was higher for participants who had set themselves a goal in the intake survey. The impact of personalization was especially observed for *frequency parameters* because the personalization of sports session frequency did foster higher engagement levels, especially when participants set a goal to improve their capabilities. In addition, the personalization of suggested ride duration had a positive effect on self-perceived biking performance.

**Conclusions:**

Personalization seems particularly promising for promoting the frequency of physical activity (eg, promoting the number of suggested sports sessions per week), as opposed to the intensity of the physical activity (eg, distance or duration). Replications and variations of our study setup are critical for consolidating and explaining (or refuting) these effects.

**Trial Registration:**

ClinicalTrials.gov NCT05264155; https://clinicaltrials.gov/ct2/show/NCT05264155

## Introduction

### Research Case

Nowadays, sedentary behavior is highly pervasive. Sedentary behavior, as distinct from physical activity, encompasses a broad range of behaviors that involve sitting or lying down and do not increase energy expenditure substantially during waking hours [1,2]. On average, adults in Western countries spend between 7 and 11 hours per day sitting [3-6]. Adults sitting >10 hours a day are expected to see their all-cause mortality rates increase [7]. Conversely, adults who participate in at least 150 minutes of moderate-intensity activity per week—an equivalent of 20 to 30 minutes per day—are expected to decrease their mortality rate significantly [8]. However, even when an adult meets these guidelines, sitting for prolonged periods can compromise health [9]. Hence, frequently interrupting periods of sitting with (short) bouts of physical activity is also essential to remain healthy [9].

Although the benefits of an active lifestyle for health are well established, it remains hard for people to engage more often in physical activity and reduce sedentary behaviors, with inactivity accounting for 9% of the premature mortality globally [10]. Mobile health (mHealth) interventions can be used to promote physical activity and reduce sedentary behavior, particularly if these tools use evidence-based behavior change strategies (eg, goal setting) [11].

Promising results have been obtained by using gamification techniques as behavior change strategies [11-13]. Gamification is a set of motivational techniques that use game mechanics outside game contexts to foster participation, engagement, and loyalty [14,15]. Gamification techniques are especially effective when they are tailored toward an individual’s particular preferences and needs (ie, personalized) [12] because behavior change techniques that motivate one person may not appeal to someone else [16]. For example, it has been demonstrated that there are significant associations between specific personality traits and the types of motivational techniques that individuals prefer [17,18], as well as the type of motivational messages that they appreciate more [19]. Furthermore, it has been suggested that interventions that take into account users’ individual capabilities when setting intervention goals are better at sustaining user engagement [20,21]. Similarly, a review of behavior change strategies to promote physical activity using mHealth interventions concluded that (adaptively) tailored goals seem to be more effective than static generic goals [22].

In this study, we aim to replicate these findings and focus on adaptively tailoring our gamified mHealth program to the capabilities of individual end users. On the basis of the findings by Sporrel et al [22], we hypothesized that an intervention that suggests health goals to its users based on the users’ capabilities and preferences will be more engaging (ie, resulting in lower dropout rates as well as higher adoption rates of healthy routines) than an intervention that does not tailor its goals. Note that in mHealth tools, capabilities are always relative to other daily routines. Specifically, the researcher is typically not interested in the participant’s actual peak capabilities for certain sports activities. Instead, a researcher typically considers the participant’s capability to perform a healthy activity in accordance with the participant’s professional and personal duties. When also considering that mHealth interventions aim to be scalable, researchers typically rely on participants’ self-reported capabilities rather than inviting all participants for an endurance test.

We aim to extend existing literature with suggestions on how goals are most effectively tailored in digital health promotion settings. Although it has already been suggested that assigned—but personalized—goals may be more effective than having users set their goals themselves [22], it remains unclear what exact strategies are most effective in setting tailored goals in a digital health promotion setting. Of course, different strategies for tailoring goals in a digital health promotion setting exist. For example, promising results have been obtained by personalizing goals based on (1) task complexity (eg, by personalizing daily step goals [23]), (2) context (eg, by setting context-aware goals [24]), or (3) the user’s autonomy to set goals (eg, by recommending goals individually instead of having users select goals from a predefined list [25]). However, the relationship between the goal target behavior (eg, to go for a walk or a run) and the impact of the goal on user engagement levels remains unclear. Hence, in this study we aim to investigate the relationship between the goal target behavior and the goal’s impact on user engagement by setting personalized goals for different types of health-related activities (ie, walking, biking, and engaging in sports).

In the following sections, we first survey the literature to examine the relationship between an individual’s capability and (suggested) goals as well as the impact of goals on behavior. Then, we detail our intervention, treatments, and study design. Subsequently, we present the results we obtained. Finally, we discuss the implications of our results and the weaknesses of this study as well as directions for future research.

### Theoretical Background

Several *behavioral* theories (eg, the COM-B [Capability, Opportunity, and Motivation Model of Behavior] System [26] and the Fogg Behavior Model [27]) argue that, for a certain (target) behavior to occur, an individual must have the *capability* and *opportunity* to engage in the (target) behavior; in addition, the strength of *motivation* to engage in it must be greater than for any competing behaviors. The concept of capability entails a person’s physical and psychological capacity to perform a target behavior [26]. Besides a person’s actual capabilities, motivation is key. Several *motivational* theories highlight that besides actual capabilities, the *perceived* ease or difficulty of performing a target behavior is an important motivating factor (ie, a concept that has been referred to as *self-efficacy* by Bandura [28] and was included as well in the Theory of Planned Behavior [29] and in Self-Determination Theory [30]).

Hence, a dilemma arises when assigning someone a behavior to perform. In particular, if the target behavior is too hard for an individual, they may feel anxious and may therefore not (continue to) engage in the behavior. In contrast, if the target behavior is too easy for them, they may feel bored and therefore may not (continue to) engage in the behavior either. Hence, an individual’s level of skill and the level of complexity (ie, challenge) of a target behavior have to be in harmony. This trade-off is very well described by Flow Theory, which was formulated by Buchanan and Csikszentmihalyi [31]. This theory has inspired the design of several (gamified) mHealth tools such as Nike+, Zombies, Run!, Fitocracy, and Runkeeper [32], all of which aim at promoting physical activity through the “provision of optimally difficult challenges and feedback” [32]. The trade-off between a person’s skill and the level of complexity of a suggested behavior is also described in Goal-Setting Theory, which proposes that task performance can be moderated by a number of factors, including task complexity and levels of self-efficacy [33]. Especially, from Goal-Setting Theory, it is known that task complexity should generally be at the verge of an individual’s capabilities to foster engagement because difficult—but specific and still attainable—tasks generally result in better performance [33].

To summarize, although tasks that are too simple lead to dropout due to boredom and tasks that are too complex trigger dropout due to anxiety (or frustration), tasks that are difficult—but specific and still attainable—generally yield the highest levels of engagement. To adhere to this principle, we designed a procedure in this study that takes into account participants’ self-reported capabilities and desired health goals in setting the tasks for them to perform.

Finally, Flow Theory points out that a person’s (perception of their) capability changes over time because their skill increases whenever they complete more challenging tasks [31]. Hence, to engage individuals in a task over a longer period time, the tasks’ complexity should be adaptively tailored in accordance with the skill they possess. For example, a recent review of behavior change strategies to promote physical activity using mHealth interventions concluded that increasing goal complexity by 20%-100% generally yields increased goal performance [22]. To adhere to this principle, we designed a procedure in this study that increased task complexity every 2 weeks to account for participants’ increased skill levels and prevent dropout due to boredom.

## Methods

### Recruitment

Participants were recruited among staff members of 7 governmental organizations (ie, 6 municipalities and 1 provincial organization) in the region of Antwerp, Belgium, in October 2019. The study was introduced to these staff members as a health promotion campaign to promote physical activity and reduce sedentary behaviors. Participants were enrolled only after they gave their explicit consent, which was collected upon registration for the campaign.

Participants were recruited by representatives of the sports departments of the participating organizations. These representatives were organized in a regional committee, with the aim to promote employee health. This committee had also called for this scientific study to be conducted. Different methods for recruiting participants were used within different organizations (ie, the means of recruiting participants were not prescribed in a study protocol). Some organizations relied on word of mouth to promote the campaign, whereas others used email advertising or printed advertisement posters. Promotional wristbands had been made available for distribution by all committee members, but we did not supervise the distribution. This approach was adopted to respect organizational differences.

### Ethical Approval

All operational procedures were approved by the ethical committee of Eindhoven University of Technology (experiment ID ERB2019IEIS5). The ethical review committee concluded that the potential benefits of this study outweighed its potential risks.

### Intervention Context

To test our hypothesis, we used the mHealth tool GameBus. GameBus was especially designed for health promotion and provides a highly configurable gamification engine that is used for sustaining participant engagement. According to the classification of gamification elements by Hamari et al [13], GameBus implements the gamification mechanisms of challenges, points, goals, progress visualizations, leaderboards, and rewards. In addition, it allows configuring of these mechanisms for testing scientific hypotheses. The tool supports hosting multiple experimental designs on a single platform, ensuring that user experience remains similar across these different designs. Moreover, the platform enables researchers to gather rich data in a manner that is compliant with European (privacy) legislations.

Using GameBus, a health promotion campaign was especially designed to promote walks, bike rides, and sports sessions. The campaign had a duration of 8 weeks and was split into 2-week periods (so-called waves).

**Figure 1:**
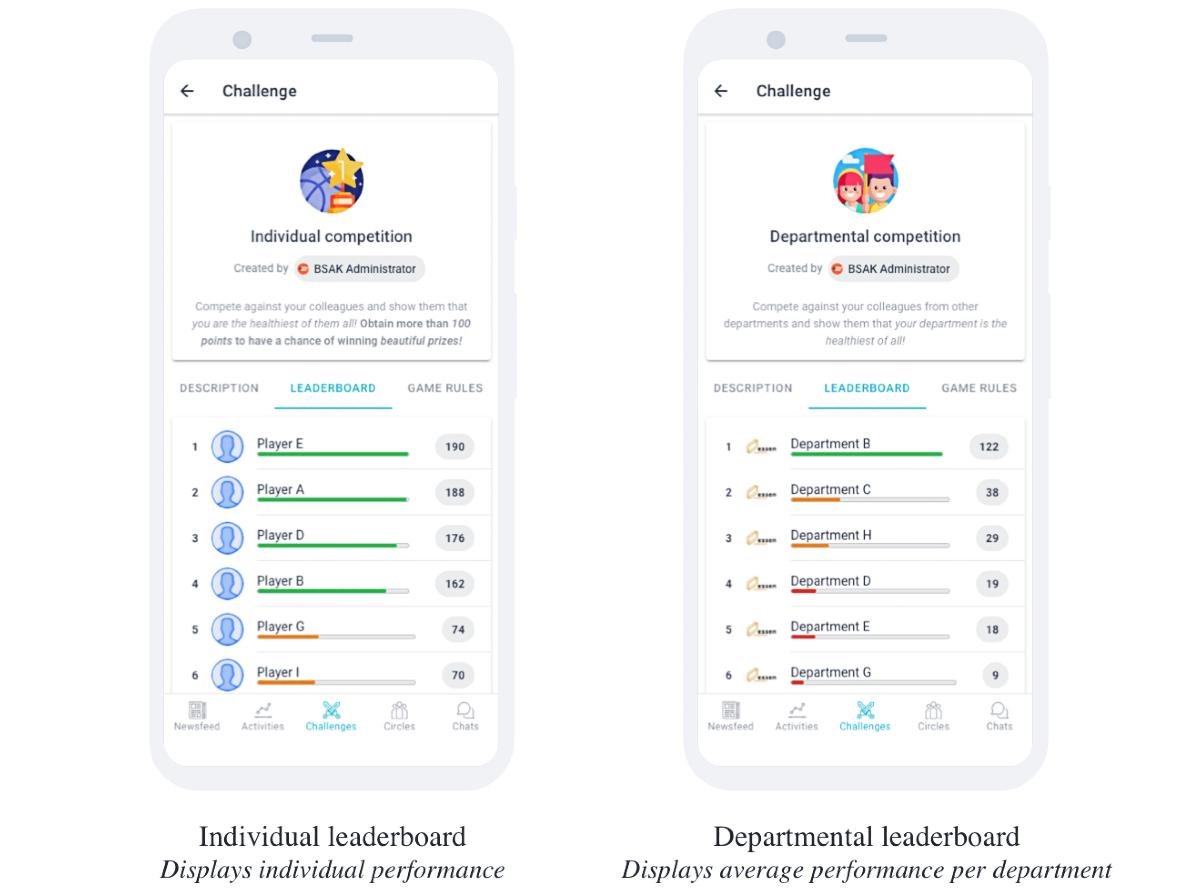
Display of social leaderboards.

To foster awareness of the campaign and stimulate word of mouth, participants could track their performance on 2 social leaderboards: a leaderboard displaying the individual scores of participants within a certain organization and a leaderboard displaying the average scores of participants within a certain municipal department. At the commencement of each wave, both leaderboards were reset (ie, scores were set back to zero). The actual implementation of both leaderboards in our mHealth tool is presented in Figure 1.

**Figure 2:**
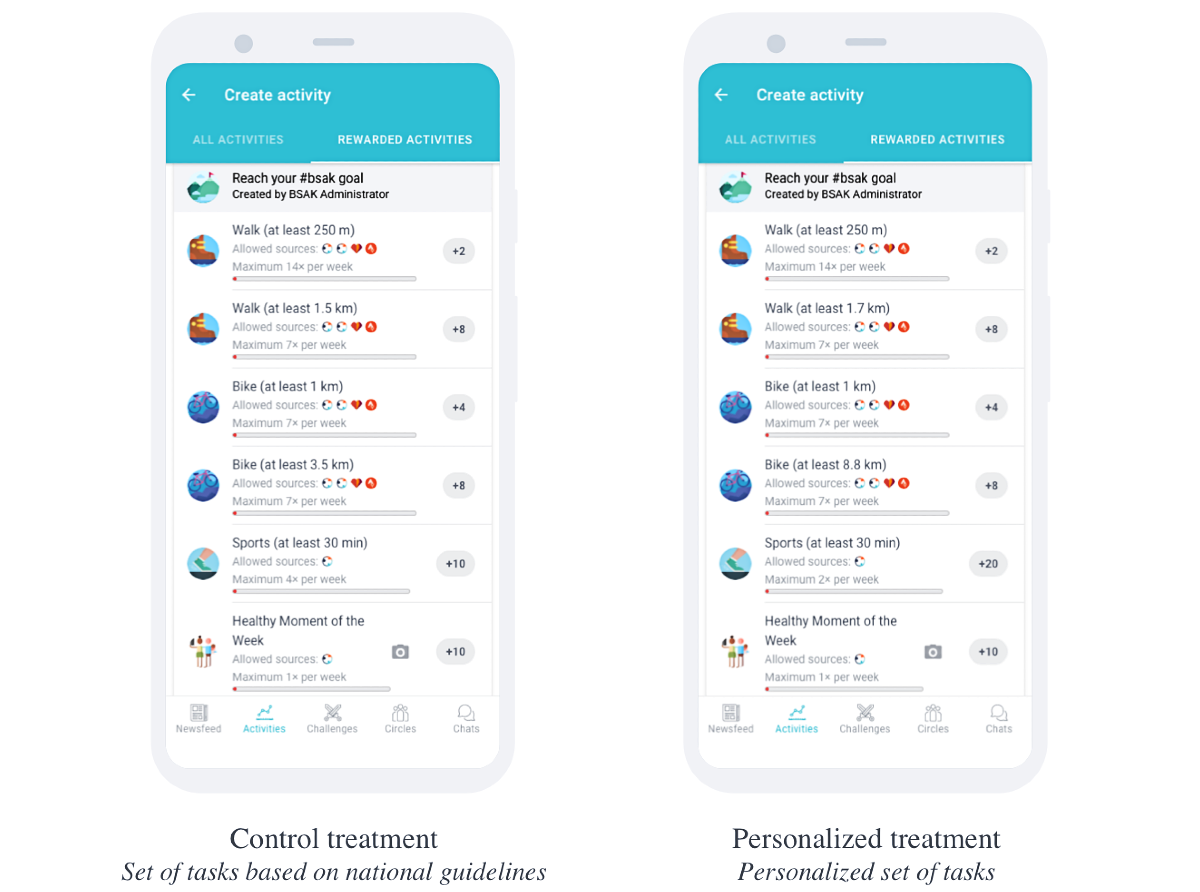
Display of the different sets of tasks per treatment.

To score points on these 2 leaderboards, a participant was given a set of tasks that, upon completion, were rewarded with points. At the commencement of each wave, a participant received a set of 6 tasks (Figure 2). The first three tasks were the same across all waves: (1) go for a short walk of at least 250 m, (2) go for a short bike ride of at least 1 km, and (3) share your healthiest moment of the week. These tasks were included to provide participants with a sense of gratification relatively easily and make them feel that *all* their physical efforts were awarded.

The other three tasks were dynamic (ie, updated at the commencement of each wave) and arguably more difficult to perform: (1) go for a longer walk of at least X km, (2) go for a longer bike ride of at least X km, and (3) go for a sports session lasting at least 30 minutes X times per week. In this study, these 3 dynamic tasks were either updated generically (ie, for the control group) or personalized based on the user’s current self-reported capabilities and health goals (ie, for the treatment group). Specific details on how these tasks were set for the different treatment groups are presented in the *Study Design* section.

Users could either manually or automatically prove their engagement with a certain task. By means of the mobile app, users could manually register that they had performed a certain task. Alternatively, users could use an activity tracker to automatically track their efforts. The activity trackers that were supported included Google Fit, Strava, and a GPS-based activity tracker that was built into the native version of the GameBus app (available for both Android and iOS devices).

To prevent users from repeating a single task over and over, we set a maximum number of points that could be obtained per task per week, as well as a maximum number of times a task was rewarded per week with points (Table 1). Note that the sports session is rewarded X times per week, where X depends on the actual campaign wave. Note that therefore the number of points awarded per sports session needs to be calculated for a given wave by dividing 40 (the maximum number of points awarded per week) by X. Figure 2 displays the exemplar sets of tasks that users in the control or treatment groups could be assigned through GameBus.

Finally, GameBus provided a set of features for social support: a newsfeed showed when other participants had scored points, and participants could *like* and comment on each other’s healthy achievements as well as chat with each other.

**Table 1:** Maximum number of points that could be obtained per suggested activity.

Task	Maximum number of points per week	Maximum number of times rewarded per week	Points per activity
Short walk	28	14	2
Longer walk	56	7	8
Short bike ride	28	7	4
Longer bike ride	56	7	8
Sports session	40	X	40/X
Healthy moment	10	1	10

### Study Design

#### Overview of Study Arms

The study was designed as a 2-arm randomized intervention trial. The experimental setup was centered around setting the complexity parameters (ie, the X values) of the 3 dynamic tasks. In particular, the parameters to determine were as follows: (1) the minimum distance of a longer walk, (2) the minimum distance of a longer bike ride, and (3) the maximum number of rewarded sports sessions (and consequently the number of rewarded points per sports session). For the control group, these parameters were based on Belgian physical activity guidelines, whereas for the personalization group, these parameters were tailored to the users’ self-reported capabilities and health goals.

#### Control Group: Tasks Based on Guidelines

For the control group, the parameter values of the dynamic tasks were based on national guidelines. The Belgian guidelines for physical activity are based on the Australian activity guidelines [34]. These guidelines recommend a minimum of 150 minutes (ie, in line with the study by Long et al [8]) of moderate-intensity activity per week, with each activity episode lasting at least 10 minutes. In addition, these guidelines suggest regularly interrupting periods of sitting with (short) bouts of physical activity (ie, in line with the study by Owen et al [9]).

On the basis of these guidelines, it was agreed with the organizing committee to suggest tasks with a duration of 10 to 30 minutes, giving participants ample opportunity to engage in at least 150 minutes of moderate-intensity activity per week. In addition, as described in Table 2, we increased the minimum durations of tasks throughout the waves by 20%-42% because it was found that increasing goal complexity (by 20%-40%) generally yields increased goal performance [35].

**Table 2:** Estimated time needed to complete a dynamic task per activity type, as suggested to the control group.

Parameter	Wave 1	Wave 2	Wave 3	Wave 4
Minimum duration of the longer walk, minutes (distance; m)	17.5 (1500)	25 (2000)	27.5 (2250)	30 (2500)
Minimum duration of the longer bike ride, minutes (distance; m)	12.5 (3500)	15 (4000)	16 (4250)	17.5 (4500)
Minimum duration of the sports session, minutes (maximum times; n)	30 (4)	30 (4)	30 (5)	30 (5)

#### Treatment Group: Personalized Tasks

To set a value for the complexity parameters of the dynamic tasks for the treatment group, it was necessary to have some insight into the users’ current capabilities and health goals. We obtained self-reports of the users’ capabilities and goals by means of a short intake survey. Note that all participants (ie, even control participants) were asked to complete this intake survey to avoid introducing a bias because just the act of declaring one’s goals may already foster motivation for the task at hand [33]. As an incentive to fill out this short survey, a donation of €0.25 (US $0.28) was made to charity for every completed survey.

In the intake survey, participants were asked to provide an estimation of (1) the number of steps they walked on a daily basis, (2) the number of kilometers they biked on a weekly basis, and (3) the number of sports sessions in which they participated on a weekly basis. Note that the participants’ capabilities were explicitly evaluated in accordance with their existing professional and personal duties because we aimed to promote health-related activities that the participants could fit in their daily routines.

Furthermore, participants were asked whether they wanted to improve on any of these (estimated) numbers. If they wanted to improve their capabilities, they were asked to express (depending on the dimension they aimed to improve) the following: (1) the number of steps they wanted to walk on a daily basis, (2) the number of kilometers they wanted to bike on a weekly basis, and (3) the number of sports sessions they wanted to attend on a weekly basis.

Subsequently, the data on participants’ capabilities and goals for walks and bike rides was transformed to fit the description templates of tasks (eg, a task has the form of *go for a longer walk of at least X kilometers*, not the form of *walk X steps per day*). The number of steps one could, and wanted to, walk per day was multiplied by 0.73 (ie, average stride length) and divided by 3 to obtain a minimum trip length (eg, to reach a goal of walking 7000 steps per day, we would suggest regularly going for a walk of at least 7000 × 0.73/3 = 1703 m). The number of kilometers one could, and wanted to, bike per week was divided by 5 (eg, to reach a goal of biking 10 km per week, we would suggest regularly going for a bike ride of at least 10/5 = 2 km).

Now we could calculate the difference between a user’s current and preferred level of capability. We would update a user’s task complexity at the commencement of each wave to linearly grow toward their goal. Hence, to personalize each parameter, we have used the formula that is displayed below, where *i* is a reference to the individual participant for whom the parameter value is calculated, *t* is the type of parameter (eg, walking distance, biking distance, or number of sports sessions), *W* is the total number of waves of the campaign (ie, 4), and *w* is the wave number of a given wave:

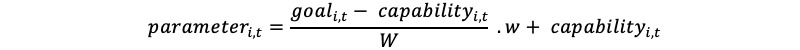



In addition, the value for *capability* was set by participants themselves (ie, by means of the intake survey). If a participant had not completed the intake survey, their *capability* was estimated to be their last performance for a particular activity type *t*. In case a participant had no recorded history on the activity type *t*, their *capability* was defined as the average performance of all other users on the activity type *t*. Note that in case there was no history of any participant on the activity type *t* yet, that *capability* was defined as a fixed value (eg, 1 km for *t* with regard to walking, 2 km for *t* with regard to biking, and 2 sessions for *t* with regard to engaging in sports).

Furthermore, a participant’s *goal* was also defined by the participants themselves, again by means of the intake survey. However, if a participant had not completed the intake survey, their *goal* was derived by multiplying their *capability* with a fixed value of 1.1 for *t* equals walks and bike rides (ie, indicating a 10% improvement) or by increasing their *capability* with a fixed value of 1 for *t* equals sports sessions.

Finally, the different parameter values were capped by a predetermined minimum and maximum. The minimum and maximum for walking distance were 1 km and 10 km, respectively; the minimum and maximum for the distance of a bike ride were 2 km and 17.5 km, respectively; and the number of sports sessions that were rewarded per week was capped between 2 and 10. For instance, if the aforementioned formula would suggest to reward 0 sports sessions, this final check would override that value, and instead allow a participant to claim points for their sports sessions twice per week.

#### Treatment Allocation

Users were allowed to join (and drop out) at any moment throughout the campaign. Whenever a user joined the campaign, they would always be given a default set of tasks until the end of the then-active wave (ie, the default set of tasks was displayed as the control treatment; Figure 2). After the wave had ended (and at the start of a new wave), a user would be allocated to either the control group or the treatment group and receive a new set of tasks accordingly.

The control and treatment samples were stratified such that each sample included the same number of people who had set a goal to improve their current capabilities (eg, new participants were immediately requested to express their current capabilities and goals through the intake survey). Obviously, the intention to improve one’s current capabilities is an important covariate because people who have a certain goal in mind are likely more motivated to engage with the campaign because this desire may influence their engagement and performance levels [33]. By stratifying our samples, the control and treatment groups were likely to be comparable.

### Study Procedures

Throughout the campaign we sent some email notifications to participants. In particular, upon registration, participants received a welcome email with a request to complete the intake survey. In addition, a campaign email was sent at the start of each wave. These campaign emails included participation instructions as well as directions for obtaining (technical) support. Finally, at the end of the campaign, a closing email with a request to fill out the posttest survey was sent. As an incentive to fill out this posttest survey, a donation of €1 (US $1.13) was made to charity for every completed survey. After 4 days, we sent out a reminder to fill out the posttest survey.

Finally, some of the 7 organizations expressed some additional requests. In particular, 1 organization (ie, the municipality of Wuustwezel) expressed the need for some additional tasks (eg, ones that were more specific than the catch-all task *Share your healthiest moment of the week*). Furthermore, the municipality of Essen requested waves with a duration of 4 weeks each (instead of a duration of 2 weeks each). For them, the social leaderboards were reset every 4 weeks (ie, twice over the entire campaign). However, note that—and this applied to the participants from Essen too—the personal set of healthy tasks was still updated every 2 weeks.

### Measurements

In mHealth, engagement is most commonly captured by means of measures of app use [36]. Using the GameBus platform, the engagement of participants was repeatedly measured as follows: (1) the number of days a participant visited the app (ie, the distinct days the participant opened the mobile app) and (2) the number of activities a participant registered. These variables complement each other because the former may be limited to passive engagement, whereas the latter requires active participation (ie, performing the suggested tasks).

Both measurements were recorded per participant per wave. In addition, for each record, the wave number relative to the participant’s participation date was recorded. Hence, a record for a particular participant who joined the campaign only in the fourth wave would have a relative wave number of zero for that record. This relative wave number was used to model time in this study to ensure that time effects (eg, novelty effects) were equal among participants.

In addition, the type of goal that the participants set in the intake survey was recorded. A participant’s goal was either *unknown* (ie, if they did not complete the intake survey), *maintain* (ie, if they did not want to improve their current capabilities on any dimension), or *improve* (ie, if they expressed an intention to improve their current capabilities on at least one dimension).

Finally, participants filled out a posttest survey (presented in Multimedia Appendix 1) in which we especially assessed the perceived impact of the campaign on their walking, biking, and sports performance, as well as their perception of their capability to perform the prescribed tasks (ie, self-efficacy).

### Statistical Analysis

The first set of statistical analyses focused on the evaluation of dropouts. A participant was labeled as a (provisional) dropout if they had not visited the app during a specific wave and was therefore assumed to have lost interest (ie, dropped out) during the previous wave. Several multiple regression models were fit to determine whether the number of dropouts changed over time and were different per treatment. In particular, we tested for significant second-order interaction effects of time (ie, the wave number) and treatment.

The second set of analyses focused on the evaluation of engagement levels of the participants. To evaluate treatment differences, further analyses were performed on participants who actually had an opportunity to receive exposure to the treatment. Hence, from the entire data set, a subset was derived preserving the combination of a particular participant and wave only if they had ever checked the app during that wave and if they had participated for a duration of at least two waves because during the wave in which a participant signed up, they were not actually receiving a treatment yet. Subsequently, several hierarchical linear models were estimated for the 2 outcome variables (ie, the number of days a participant visited the app and the number of activities a participant had registered) using time (ie, the relative wave number), participant’s goal, and treatment as predictors. We tested whether significant second-order interaction effects existed among these variables. In all models we allowed random intercepts for both individuals and the governmental organizations they were part of. The final model was selected based on the Akaike information criterion [37]. The Akaike information criterion estimates the relative quality of statistical models for a given set of data. The measure rewards goodness of fit and includes a penalty for increasing the number of predictors (ie, to prevent overfitting because increasing the number of predictors generally improves the goodness of the fit).

In addition, a third set of analyses zoomed in on the experimentally controlled tasks (ie, the longer walk, the longer bike ride, and the sports sessions) to evaluate treatment differences at the level of individual activity types. Specifically, for each activity type, a hierarchical linear model was built to predict the number of times a participant registered a task for that particular activity type. Again, these models included time (ie, the relative wave number), participant’s goal, and treatment as predictors. In addition, we tested whether significant second-order interaction effects existed among these variables. In all models we allowed random intercepts for both individuals and the governmental organizations they were part of. The final model was again selected based on the Akaike information criterion [37].

Finally, the fourth set of analyses focused on the evaluation of subjective measures that were derived from a posttest survey. This final set of analyses was performed on a subset of the data set that only included participants who filled out the posttest survey and were using the mHealth app in more than one wave. A set of 3 separate linear models was used to estimate the perceived impact of the campaign on walking performance, biking performance, and sports performance. An additional linear model was used to estimate participants’ perception of their capability to perform the tasks they were prescribed (ie, self-efficacy). Again, in all 4 models, time (ie, the total number of waves a participant had been visiting the app), participant’s goal, and treatment were used as predictors, and we tested whether significant second-order interaction effects existed among these variables. To obtain the final models, a backward elimination selection procedure was used [38]. Backward elimination starts with all predictors included in the model, with variables subsequently being eliminated one at a time. At each step, the predictor with the highest *P*>.05 is deleted [38]. This method of deletion continues until all predictors are significant (ie, *P*<.05).

### Ethics Approval

All operational procedures were approved by the ethical committee of Eindhoven University of Technology (experiment ID ERB2019IEIS5). The ethical review committee concluded that the potential benefits of this study outweighed its potential risks.

## Results

### User Statistics

**Figure 3:**
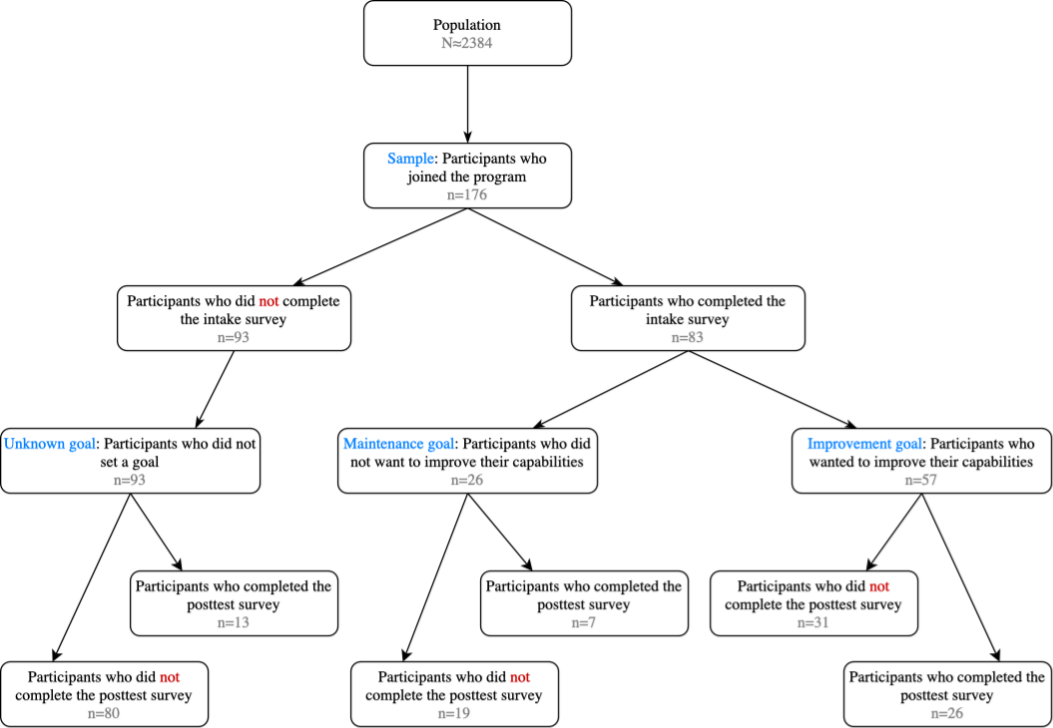
Cohort diagram that details the number of participants engaged in different study phases.

In total, 176 unique participants joined the study, and they were randomly assigned to a treatment: 82 (46.6%) were assigned to the control treatment and 84 (47.7%) were assigned to the personalized treatment, whereas 10 (5.7%) were not assigned to a treatment at all because they only signed up during the last wave and therefore only experienced the default set of tasks. Of the 176 participants, 83 (47.2%) completed the intake survey (26/83, 31%, set themselves a maintenance goal and 57/83, 69%, set themselves an improvement goal), whereas 93 (52.8%) did not complete the intake survey and hence their goal was unknown. These data are summarized in Figure 3, which displays a cohort diagram that details the number of participants engaged in different study phases.

Table 3 displays sample demographics based on the results of the posttest survey, which was filled out by 26.1% (46/176) of the participants. Gender, age group, and personality scores are displayed for the entire sample as well as per treatment. The demographic characteristics in the control group and treatment group are distributed similarly. Hence, it is assumed that these groups were comparable at baseline.

**Figure 4:**
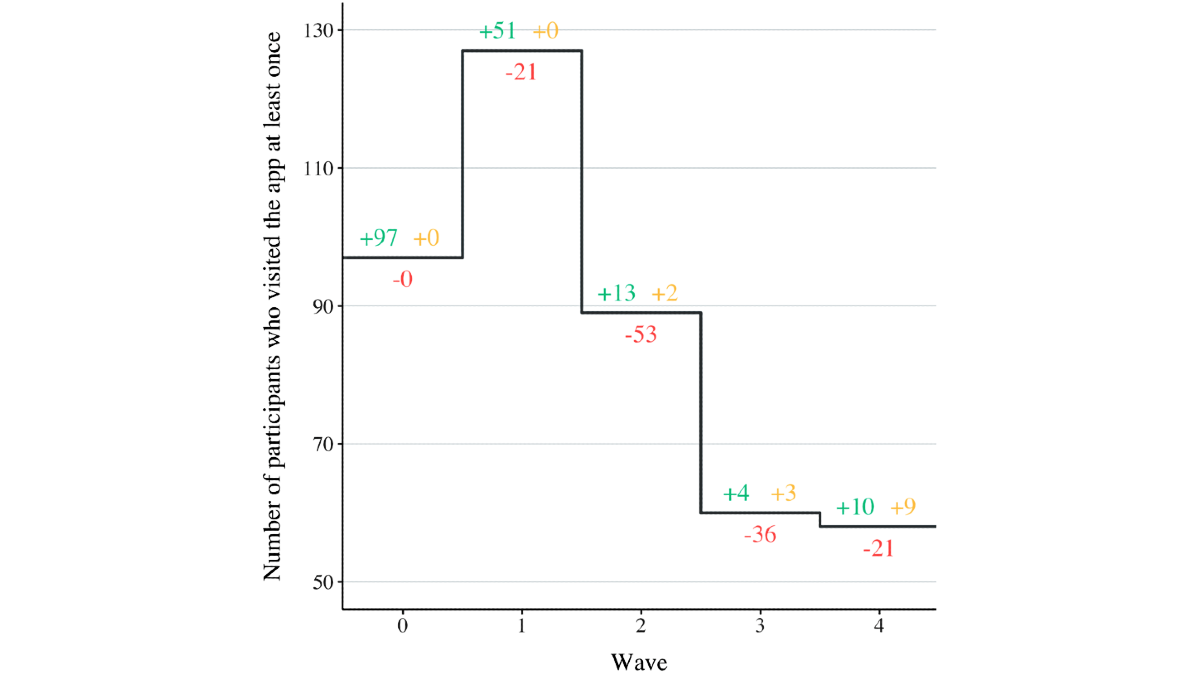
Number of participants who visited the app at least once per wave.

Figure 4 displays the decrease in the number of participants who visited the mobile app during a given wave. The number of participants who joined the campaign for the first time during a given wave are displayed in green. The number of participants who dropped out during a specific wave are displayed in red. The number of participants who checked the mobile app during a specific wave, although they dropped out during an earlier wave (ie, reclaimed users) are displayed in yellow. Using multiple regression analysis, it was found that participants tended to drop out over time (ie, the wave number is a significant factor for predicting dropouts at *P*=.03; Table S1 in Multimedia Appendix 2). No significant differences in dropout rates between treatments could be detected. In addition, no significant interaction effect between time (ie, the wave number) and treatment was detected. Hence, it is assumed that dropouts were spread equally over treatments.

**Table 3:** Sample demographics (N=46).

Characteristic		Sample, n (%)	Control group, n (%)	Treatment group, n (%)	No treatment, n (%)
**Gender (n=46)**					
	Male	13 (28)	7 (54)	5 (38)	1 (8)
	Female	33 (72)	15 (45)	18 (55)	0 (0)
**Age group (years; n=44)**					
	21-30	12 (27)	6 (50)	5 (42)	1 (8)
	31-40	16 (36)	7 (44)	9 (56)	0 (0)
	41-50	3 (7)	2 (67)	1 (33)	0 (0)
	51-60	8 (18)	4 (50)	4 (50)	0 (0)
	61-70	5 (11)	1 (20)	4 (80)	0 (0)
**Personality scores (n=41),** *μ* **,** *σ*					
	Openness	2.573, 0.610	2.500, 0.473	2.643, 0.723	—^a^
	Conscientiousness	2.329, 0.616	2.250, 0.579	2.405, 0.654	—
	Extraversion	2.780, 0.645	2.763, 0.599	2.798, 0.701	—
	Agreeableness	2.006, 0.476	1.975, 0.499	2.036, 0.463	—
	Neuroticism	3.299, 0.710	3.362, 0.681	3.238, 0.748	—

^a^Posttest personality scores were not available for the participant who was not assigned a treatment.

### Descriptive Statistics of Complexity Parameters

The complexity parameters of the dynamic tasks that the control participants were assigned are presented in Table 2. However, the complexity parameters for the treatment group were different for each individual in that group and were only determined at the start of a new wave. The mean (SD), minimum, and maximum values of the 3 complexity parameters are displayed per wave in Table 4.

**Table 4:** Mean (SD), minimum, and maximum values of the complexity parameters per dynamic task as presented to the treatment group.

Parameter			Wave 1		Wave 2		Wave 3		Wave 4
**Minimum distance of the longer walk (m)**									
	Mean (SD)	1777 (600)		2025 (500)		2091 (488)		2054 (526)	
	Minimum	1000		1000		1000		1004	
	Maximum	4365		4471		4578		4684	
**Minimum distance of the longer bike ride (m)**									
	Mean (SD)	7822 (3704)		7439 (3194)		7718 (3386)		9127 (3728)	
	Minimum	2000		2000		2000		2000	
	Maximum	17,500		17,500		17,500		17,500	
**Suggested sports sessions**									
	Mean (SD)	2.41 (1.02)		2.97 (0.83)		3.67 (0.85)		3.09 (0.93)	
	Minimum	2		2		2		2	
	Maximum	7		7		7		8	

### Evaluation Outcomes

#### Evaluation of Engagement Levels

##### Description of the Data Set

Of the 176 participants, 10 (5.7%) only joined the study during the last wave; hence, they were not assigned a treatment and were therefore excluded from further statistical analysis, leaving 166 (94.3%) participants in the data set. In addition, of these 166 participants, 55 (33.1%) only visited the app at their registration (ie, during their first wave) and hence were also excluded from further statistical analysis, leaving a total of 111 (66.9%) participants in the data set for evaluation of engagement levels (ie, 51/111, 45.9%, assigned to the control treatment and 60/111, 54.1%, assigned to the personalized treatment).

##### Impact on Passive Engagement Levels

**Figure 5:**
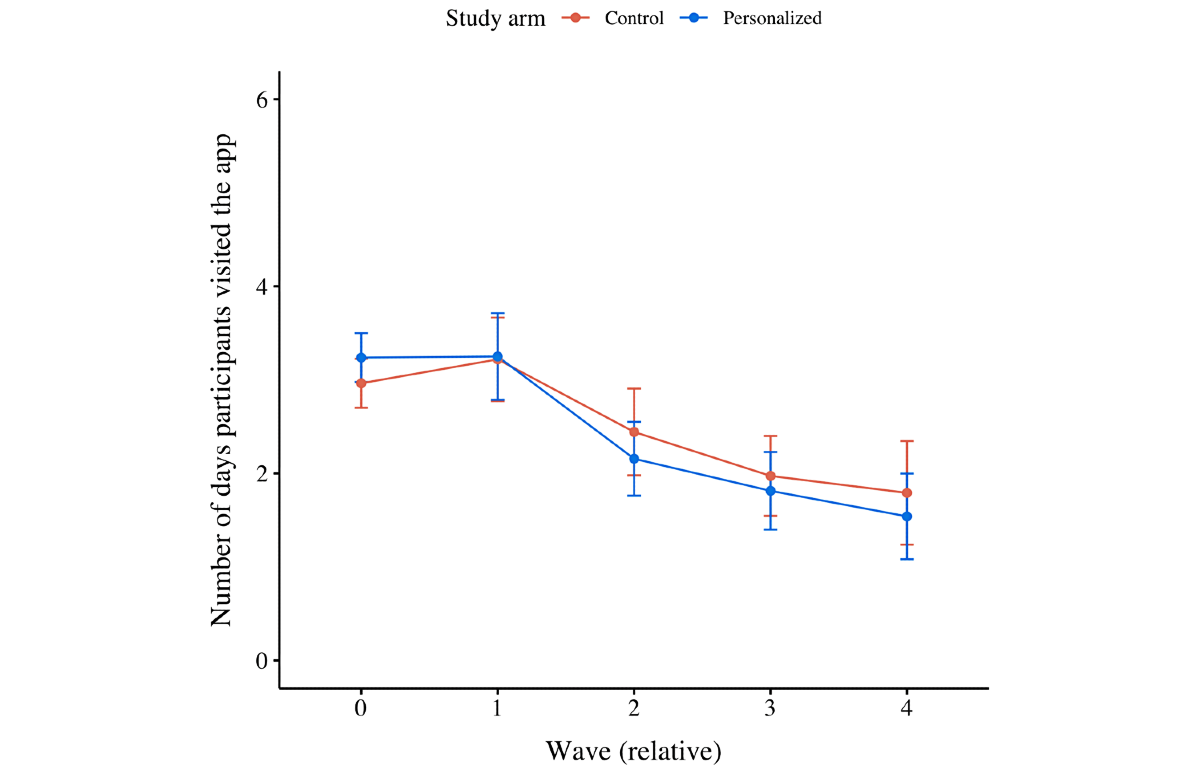
Mean plot of the number of days participants visited the app per wave, per treatment.

**Figure 6:**
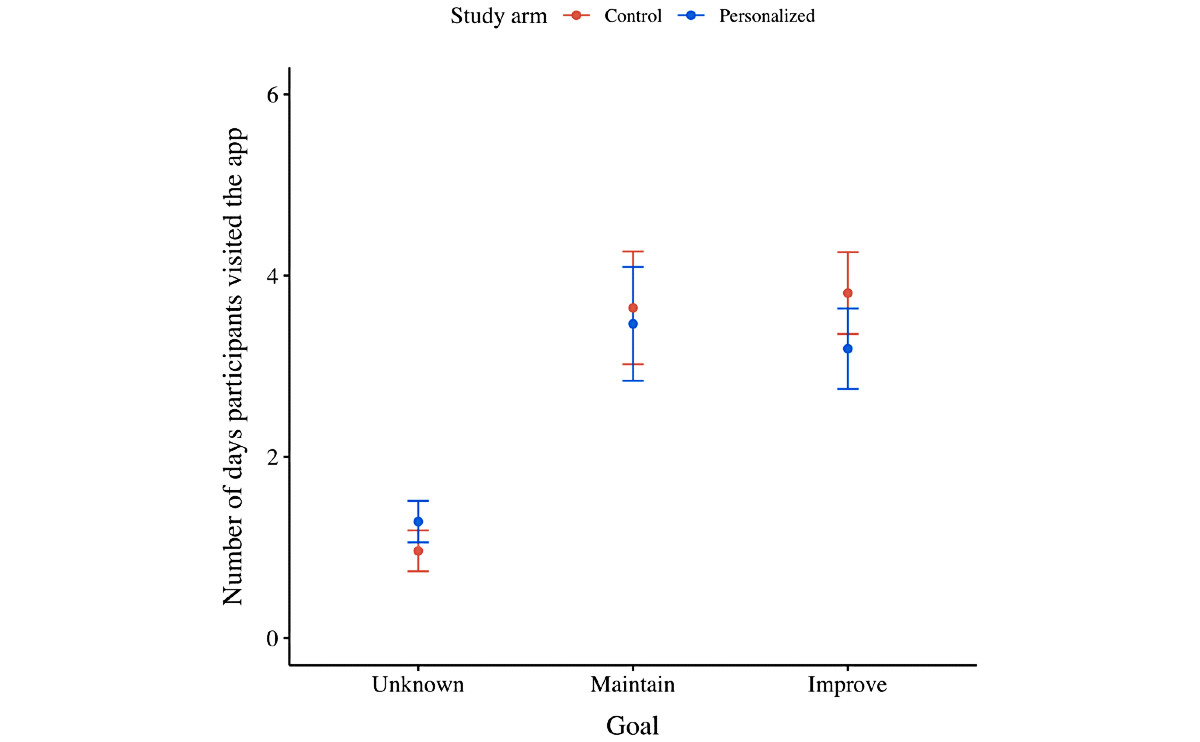
Mean plot of the number of days participants visited the app per their ambition to improve their current capabilities, per treatment.

Figure 5 displays the number of days participants visited the app on average per wave per treatment. Figure 6 displays the number of days participants visited the app on average per type of goal they set.

From the second set of statistical analyses, it was found that the number of days participants visited the app dropped over time (ie, –1.174 days per relative wave; *P*<.001; Figure 5 and Table S2 in Multimedia Appendix 2). No significant difference between treatments was detected, although it did matter whether participants completed the intake survey. In particular, participants who completed the intake survey—and hence set themselves a goal to either maintain or improve their current capabilities—visited the app on more distinct days than those who did not set themselves a goal (ie, +2.176 days for participants with a maintenance goal; *P*<.001; and +1.625 days for participants with an improvement goal; *P*=.005; Figure 6). Finally, no significant interaction effects were detected; all treatments were affected equally by the impact of time.

##### Impact on Active Engagement Levels

**Figure 7:**
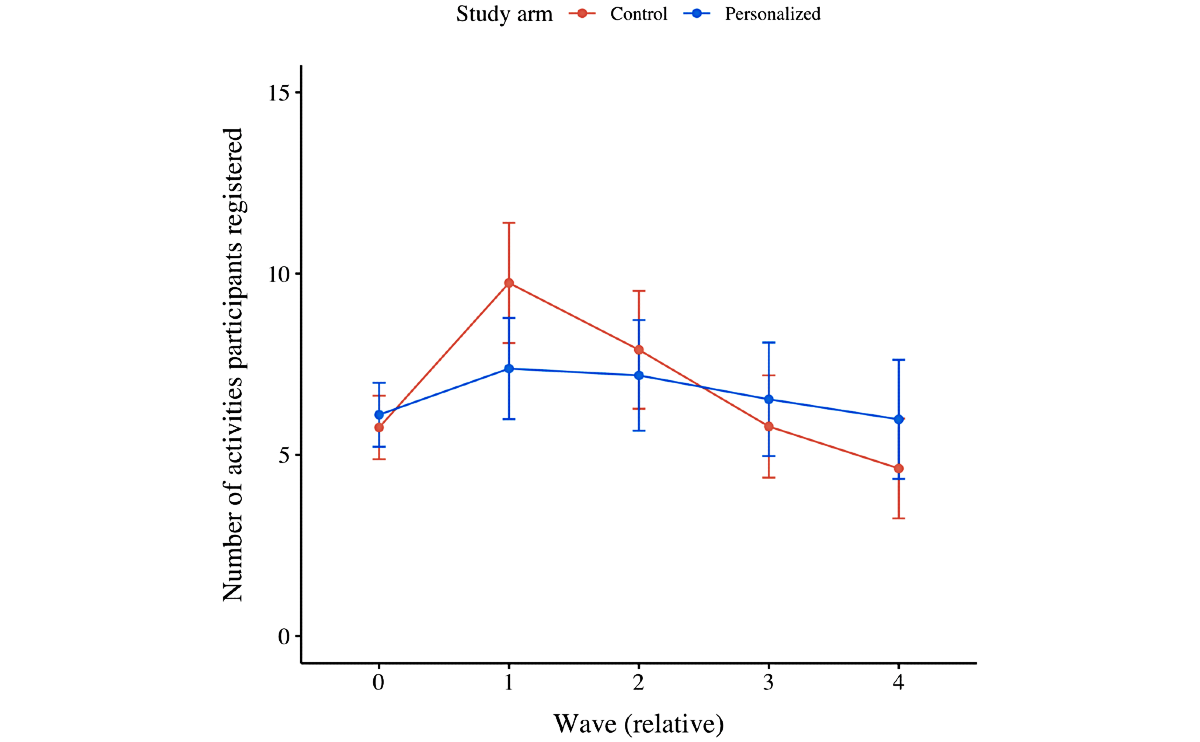
Mean plot of the number of activities participants registered per wave, per treatment.

**Figure 8:**
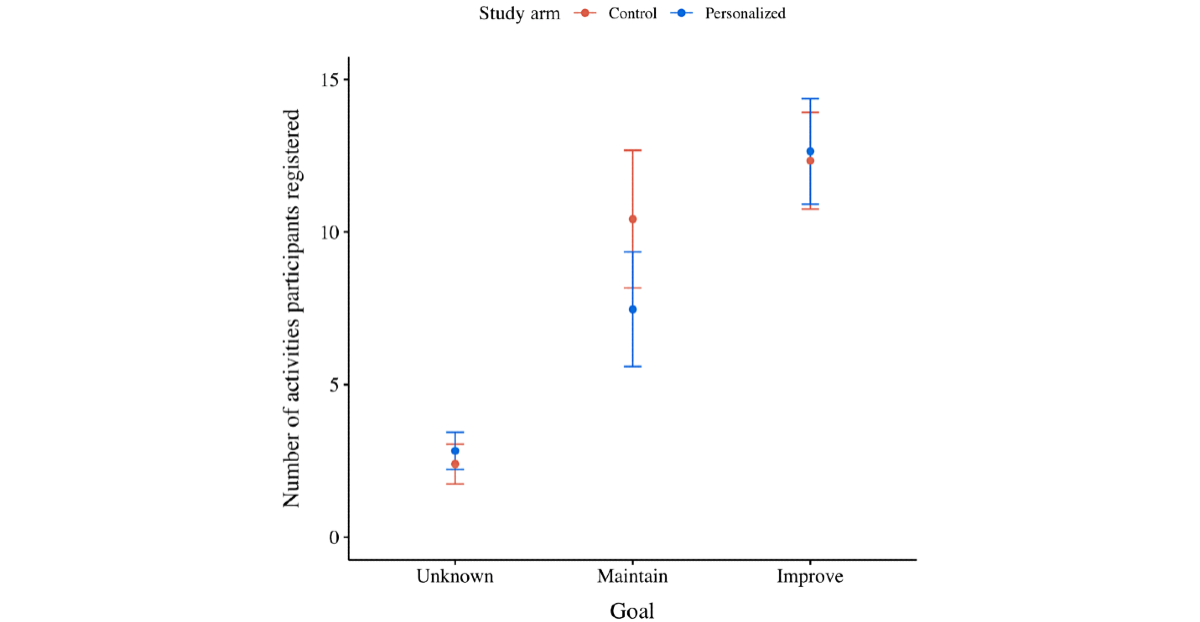
Mean plot of the number of activities participants (per treatment) registered after they were grouped based on their ambition to improve their current capabilities.

Figure 7 displays the average number of activities participants registered per treatment. Figure 8 displays the average number of activities participants registered per type of goal they set. Multimedia Appendix 3 displays an overview of the number of times a particular suggested task was registered per organization.

Moreover, from the second set of statistical analyses, it was found that the number of activities participants registered decreased over time (ie, –0.080 activities per wave; *P*<.001; Figure 7 and Table S3 in Multimedia Appendix 2). No significant difference between treatments was detected, although it did matter whether participants completed the intake survey. In particular, participants who set themselves a maintenance goal registered more activities than those who did not set themselves a goal (ie, +1.535 activities; *P*=.03; Figure 8). Moreover, participants who set themselves an improvement goal registered even more activities (ie, +3.258 activities; *P*<.001; Figure 8). Finally, no significant interaction effects were detected; again, all treatments were affected equally by the impact of time (ie, relative wave number).

##### Impact on the Execution of Particular Activities

**Figure 9:**
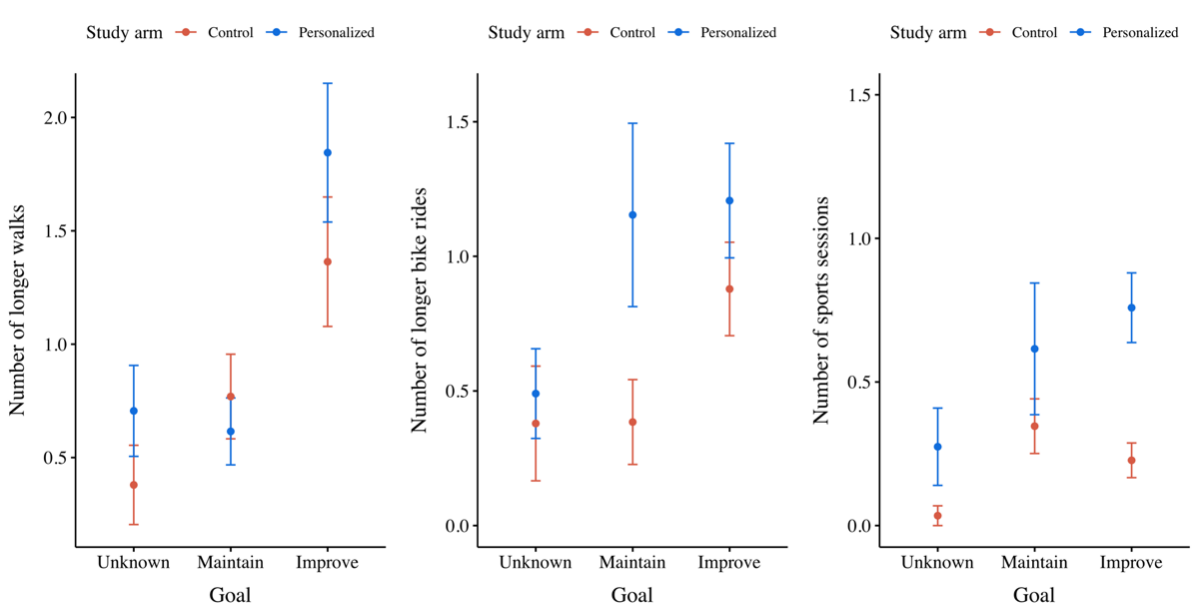
Mean plots of the number of longer walks, longer bike rides, and sports sessions participants registered per ambition to improve their current capabilities, per treatment.

The third set of analyses zoomed in on the experimentally controlled tasks (ie, the longer walk, the longer bike ride, and the sport sessions) to evaluate treatment differences at the level of individual activity types (Figure 9). For each activity type, a hierarchical linear model was built to predict the number or times a participant registered a task for that particular activity type. No significant predictors were found for estimating the number of longer bike rides a participant registered. However, the number of longer walks a participant registered depended particularly on the goal they had set (ie, +0.261 walks for maintenance goals; *P*=.53; and +0.917 walks for improvement goals; *P*=.004; Table S4 in Multimedia Appendix 2). Moreover, the number of sports sessions a participant registered was dependent not only on the goal they had set (ie, +0.405 sports sessions for maintenance goals; *P*=.05; and +0.318 sports sessions for improvement goals; *P*=.04), but also on the treatment they had been assigned to (ie, +0.276 sports sessions if personalized; *P*=.05; Table S5 in Multimedia Appendix 2).

#### Perception Analysis

##### Description of the Data Set

Finally, we analyzed the participants’ perception of their performance as well as capability to complete the program’s suggested tasks (ie, self-efficacy). This fourth set of analyses was performed on a subset of the data set that only included participants who (1) filled out the posttest survey and (2) were using the mHealth app in >1 wave. This resulted in a data set of 38 participants (ie, 20, 53%, assigned to the control treatment and 18, 47%, assigned to the personalized treatment).

##### Perceived Impact on Performance

**Figure 10:**
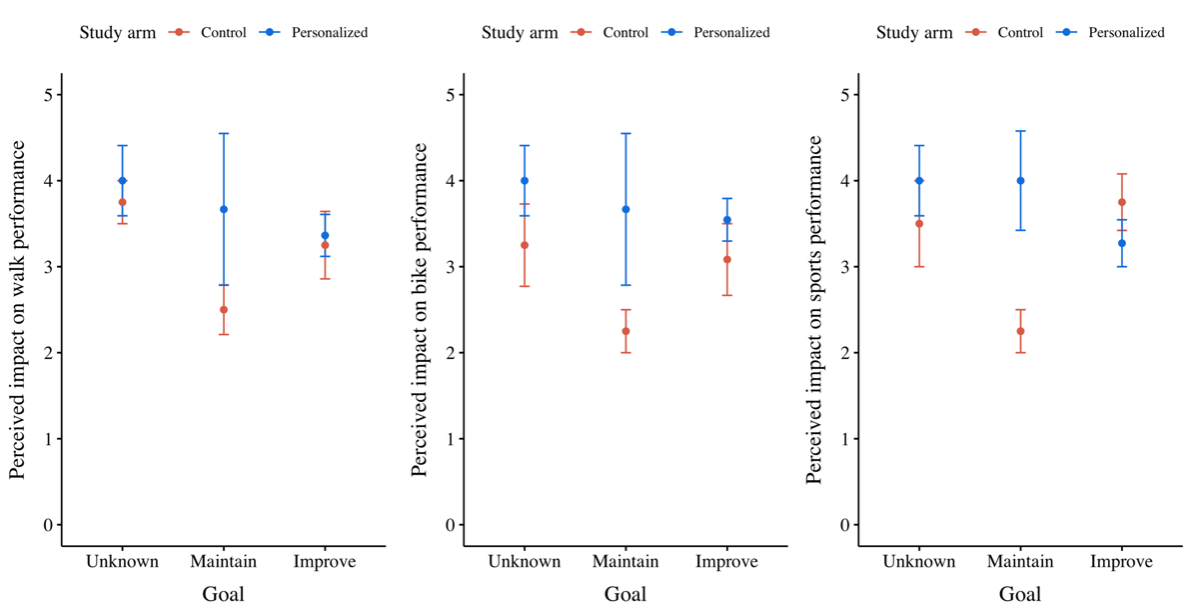
Mean plots of the perceived impact on walk, bike, and sports performance per participants’ ambition to improve their current capabilities, per treatment.

When zooming in on the perceived impact on performance of individual activity types (ie, walks, bike rides, and sports sessions), no significant predictors were found for estimating the perceived impact on walk performance (Figure 10). Nevertheless, the perceived impact on bike performance depended particularly on the treatment a participant received (ie, +0.047 if personalized; *P*=.04; Table S6 in Multimedia Appendix 2). In addition, the perceived impact on sports performance was dependent on a significant interaction effect between the treatment a participant received and the goal they had set (ie, +0.500 if personalized and a goal to maintain their current capabilities and –0.227 if personalized and a goal to improve their performance; *P*=.04; Table S7 in Multimedia Appendix 2).

##### Impact on Perception of Capability

**Figure 11:**
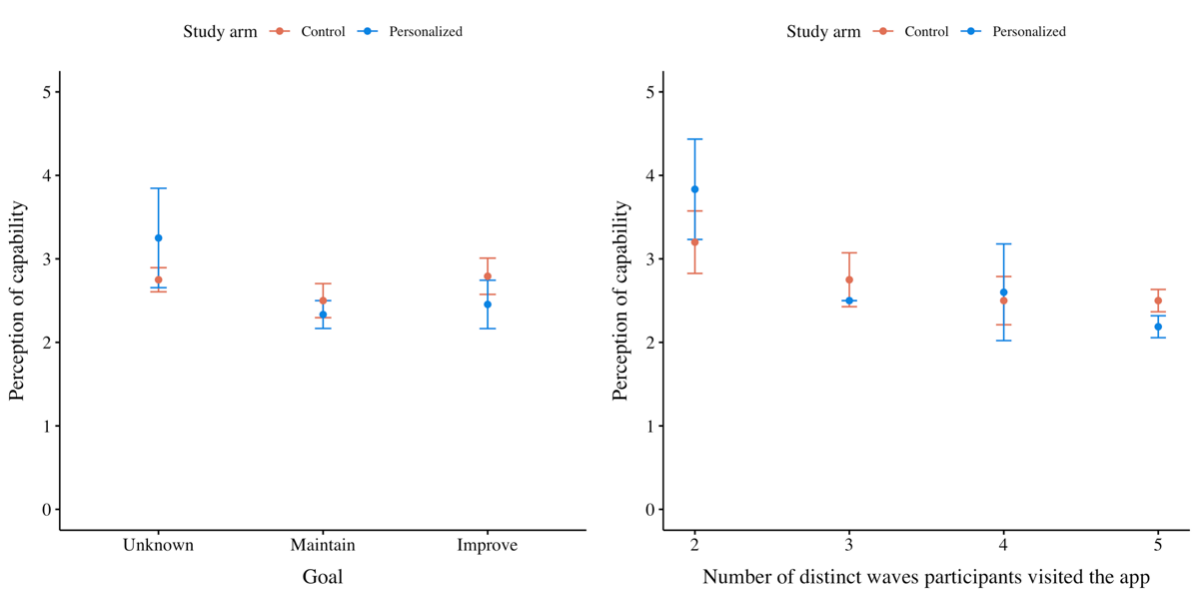
Mean plots of the perception of capability (ie, self-efficacy) per treatment. The chart on the left groups participants based on their ambitions to improve their current capabilities, and the chart on the right groups participants based on the number of waves during which they remained active.

Finally, the fourth set of analyses yielded a linear model to estimate the participants’ perception of their capability to perform the prescribed activities (ie, self-efficacy; Figure 11). The treatment did not have a significant impact on the participants’ perception of their capability. Nevertheless, for both the control and treatment groups, the perception of capability diminished over time (ie, –0.329; Table S8 in Multimedia Appendix 2) because the parameter measuring the total number of waves in which a participant had been visiting the app was reported significant at *P*=.001.

## Discussion

### Principal Findings

The aim of this study is to evaluate the impact of personalized goal setting in a gamified health promotion program on participant engagement levels. Our results show that engagement with the program inevitably dropped over time, both in the personalized condition and in the control condition. Although this pattern is common in digital health promotion programs [39], several factors may be relevant for explaining this tendency in this particular context. First, it must be noted that only a limited number of participants had explicitly set a goal to maintain or improve their current capabilities (ie, 83/176, 47%). According to the Transtheoretical Model of Behavior Change, there are 5 sequential *Stages of Change* that characterize one’s readiness for change [40]. Hence, a great proportion of our sample seemed to be still in the precontemplation or contemplation phase, phases in which they were actually not (yet) planning for a more active lifestyle. Second, it must be noted that the participants’ autonomy was limited during this program (eg, they were not rewarded for improving their dietary intake but instead only received suggestions for improving their levels of physical activity), which—according to Self-Determination Theory—may have harmed their intrinsic motivation levels [30].

Still, the participants who had set themselves a goal (ie, by completing the intake survey) were more engaged than those who had not. In particular, these participants visited the app more frequently and also registered more of the healthy tasks they were prescribed. Hence—as proposed by Goal-Setting Theory—setting a goal is in itself a motivating task [33]. Nevertheless, improvement goals—which are arguably more difficult to achieve than maintenance goals—did not seem to be significantly more motivating in general than maintenance goals. This finding seems to contradict both Flow Theory and Goal-Setting Theory, which propose that difficult—but still attainable—goals are more engaging than easier goals [31,33]. Then again, it should be noted that the descriptive means were mostly in the expected direction (ie, improvement goals were more engaging than maintenance goals) and the impact of improvement goals was actually significantly larger for promoting sports sessions: if a participant explicitly expressed a need to improve their current performance, they perceived their sports performance to be improved significantly.

Finally, the impact of the personalized treatment on engagement levels seemed to be generally limited. However, descriptive means were mostly in the expected direction (ie, personalized goals were more engaging than generically suggested goals). The seemingly limited impact of personalized goal setting may be explained by the actual strategy for personalizing the set of tasks. Moreover, we found that personalizing the suggested minimum number of sports sessions did stimulate participants to perform significantly more sports sessions, as well as significantly improved their perception of their sports performance. Upon close examination of this complexity parameter, we found that it can be characterized as a frequency parameter, whereas the parameters for personalizing walks and bike rides are typically characterized as intensity parameters. A frequency parameter defines *how many times* a particular activity should be performed in a given time frame, whereas an intensity parameter defines *how* a particular activity should be executed (eg, for how long and how far). We are unaware of context-specific factors that could have influenced this effect. However, we cannot claim generalizability yet either.

Finally, it must be noted that the treatment group participants did not feel more capable of completing the program’s tasks than the participants in the control group. Although no significant differences between the treatment groups could be detected with respect to the participants’ perception of capability to complete the program’s tasks (ie, self-efficacy), the treatment group participants who set themselves a goal reported the lowest levels of self-efficacy on average among all participants. Hence, our personalization strategy may have suggested tasks that were perceived as too difficult or too easy by our target users, thereby potentially compromising self-efficacy and engagement with the program [30,31].

### Limitations

The execution of this study was subject to several limitations. First, participants could take part without completing the intake survey. As a result, it was unknown in the case of some participants whether they explicitly choose not to set goals for themselves or whether they actually did aim to maintain or improve their current capability levels.

Second, participants may have felt that the number of points they were awarded for their activities, which affected their position on the social leaderboard, was unfair. By nature of the personalized treatment, each participant’s intervention program was unique (ie, the intervention program was tailored to participants’ individual capabilities and goals). Although, objectively speaking, this tailoring strategy makes the whole competition actually more fair, we received reports from several participants perceiving it as unfair that they had to (seemingly) expend more effort than their colleagues to be awarded the same number of points.

Third, an additional design choice that participants may have perceived as unfair was the decision to reward walks and bike rides on a per-trip basis, instead of, for example, on a daily aggregate basis. As a result, participants who went out for multiple shorter walks may not have been sufficiently rewarded for their effort. Then again, our aim was to promote activities with a minimum duration of 10 minutes, but perhaps it is worthwhile exploring this trade-off in more depth.

Fourth, the study outcomes were largely based on self-reported measures. Although participants could automatically (ie, objectively) prove their engagement with a certain task using Google Fit, Strava, or a built-in GPS-based activity tracker, they were also allowed to manually (ie, subjectively) claim that they had engaged in a certain task. This design choice could have introduced fraudulent activity registrations.

Fifth, the posttest survey suffered from low response rates (ie, 46/176, 26%). This low response rate on the posttest survey may have introduced a selection bias in the fourth set of analyses of subjective measures.

Finally, this study evaluated the impact of our intervention on a particular target group (ie, government staff) within a specific context (ie, the work environment). It is likely that the results will be generalizable to other audiences and contexts—because both Flow Theory [31] and Goal-Setting Theory are universal theories [33]—but it remains unclear what the intervention’s exact impact on health behavior would be in different settings.

### Future Work

A follow-up study should better control how participants set goals for themselves (ie, by means of the intake survey). For example, participants could be required to complete the intake survey before they are allowed to engage in the (gamified) program. Moreover, the intake survey could be extended to also assess participants’ Stage of Change according to the Transtheoretical Model of Behavior Change [40]. It seems natural to set different goals for participants who are in the precontemplation or contemplation phase (ie, the phase in which participants are not [yet] planning for a more active lifestyle) and for participants who are already actively improving their lifestyle (ie, participants in the action phase). Perhaps these 2 groups need to be assigned a different (gamified) program altogether.

In addition, future work should focus on evaluating different strategies for personalizing goal parameters. A particular opportunity is exploring in more detail the potential impact of personalizing the frequency parameters, rather than the intensity parameters. Focusing on promoting activity frequency particularly satisfies physical activity guidelines, which suggest that frequently interrupting periods of sitting with (short) bouts of physical activity is essential to remain healthy because sitting for prolonged periods can in itself compromise health [9]. Does personalization based on frequency parameters also have a larger impact on engagement levels in general? And if so, why? Finally, future work could explore the impact of allowing participants to add personalized goals for other types of activities too (eg, healthy dietary intake).

### Recommendations

Although we have not yet been able to generalize our findings to support the claim that personalizing activity frequency fosters engagement levels better than personalizing activity intensity, we still suggest that practitioners focus on setting personalized goals based on activity frequency, in particular, because focusing on activity frequency implies performing physical activity more often (instead of for longer duration or performing more intense physical activity). This focus adheres especially well to physical activity guidelines, which suggest that frequently interrupting periods of sitting with (short) bouts of physical activity is essential to remaining healthy because sitting for prolonged periods can in itself compromise health [9]. Meanwhile, we encourage scholars to replicate our study setup to gain a deeper understanding of the potential impact of different strategies for tailoring health goals. To this end, we recommend that scholars (also) apply Goal-Setting Theory [33] and Flow Theory [31] when designing their studies. Similarly, we encourage scholars to evaluate the relationship between strategies of adaptive goal setting and contextual factors (eg, whether outcomes can be replicated with other target audiences).

### Conclusions

In this study, we evaluated a gamified program that was designed to promote engagement in physical activity with sedentary government staff. Our aim is to investigate the impact of adaptive goal-setting strategies on end-user engagement levels with the program. In particular, through the program, study participants were stimulated to engage in a set of health-related activities (eg, to go for a walk, run, or sports session). Of these activities, we tailored the suggested intensity (ie, the minimum walking or biking distance) and frequency (ie, for sports sessions) based on the end users’ self-reported current capability (eg, current walking capability) and desired capability (eg, desired walking capability). Our results indicated that end-user engagement with the program inevitably decreased over time. However, compared with a control group, it was found that tailoring the frequency of suggested activities (ie, as opposed to tailoring the intensity of activities) does promote engagement in that activity (ie, engaging in sports sessions). This effect was reported to be especially strong in participants who expressed an intention to improve their health-related capabilities at the beginning of the program. In fact, engagement was generally higher in participants who expressed an intention to improve their capabilities on at least one health dimension. Hence, when designing a gamified health promotion program, end-user engagement levels may be fostered by having end users explicitly state their current and desired capabilities and by setting health goals that tailor the suggested frequency of engaging in activities that constitute these goals.

## Appendix

Multimedia Appendix 1 Posttest survey.

Multimedia Appendix 2 Detailed output of statistical analysis.

Multimedia Appendix 3 Overview of the number of times a particular suggested task was registered per organization.

Multimedia Appendix 4 CONSORT eHealth Checklist (v1.6.1).

## Abbreviations

COM-B: Capability, Opportunity, and Motivation Model of Behavior
mHealth: mobile health
