# Supplementary material for: Evaluating the Impact of Adaptive Personalized Goal Setting on Engagement Levels of Government Staff With a Gamified mHealth Tool: Results From a 2-Month Randomized Controlled Trial
Source: JMIR Mhealth Uhealth. 2022 Mar 31;10(3):e28801. doi: 10.2196/28801 (PMC9015741; doi:10.2196/28801)
Supplement: Multimedia Appendix 1 [file mhealth_v10i3e28801_app1.pdf]

Evaluation of the health promotion program ‘Beweeg & Scoor’

Personal information

1. What is your email address?
2. Which municipality / organization do you work for?
    - Brasschaat
    - Essen
    - Kapellen
    - Schoten
    - Stabroek
    - Wuustwezel
3. Which department do you work for?
4. What is your gender?
    - Male
    - Female
    - Undisclosed
5. What is your age?
    - 00 - 20 years old
    - 21 - 30 years old
    - 31 - 40 years old
    - 41 - 50 years old
    - 51 - 60 years old
    - 61 - 70 years old
    - 71 - 99 years old

Activities & virtual points

6. I enjoyed the activities that were awarded virtual points in GameBus.
    - Strongly agree
    - Agree
    - Neutral
    - Disagree
    - Strongly disagree
7. I was able to perform the activities that were awarded virtual points in GameBus.
    - Strongly agree
    - Agree
    - Neutral
    - Disagree
    - Strongly disagree
8. The number of virtual points that were awarded in GameBus for performing different activities was evenly distributed.
    - Strongly agree
    - Agree

- Neutral
- Disagree
- Strongly disagree

9. I believe that my colleagues did not cheat.
   - Strongly agree
   - Agree
   - Neutral
   - Disagree
   - Strongly disagree
10. I felt that the activities that were awarded virtual points in GameBus were at my level.
   - Strongly agree
   - Agree
   - Neutral
   - Disagree
   - Strongly disagree
11. Throughout the campaign I felt especially related to my colleagues.
   - Strongly agree
   - Agree
   - Neutral
   - Disagree
   - Strongly disagree

Health impact

12. I went for a **walk** more often than usual due to the Beweeg & Scoor program.
   - Strongly agree
   - Agree
   - Neutral
   - Disagree
   - Strongly disagree
13. I went for a **bike ride** more often than usual due to the Beweeg & Scoor program.
   - Strongly agree
   - Agree
   - Neutral
   - Disagree
   - Strongly disagree
14. I engaged in **sports sessions** more often than usual due to the Beweeg & Scoor program.
   - Strongly agree
   - Agree
   - Neutral
   - Disagree
   - Strongly disagree
15. I have been in touch with colleagues more often than usual due to the Beweeg & Scoor program.

- Strongly agree
- Agree
- Neutral
- Disagree
- Strongly disagree

Automatic registration of activities

16. Were you aware of the possibility to have GameBus automatically record your physical activities for you?
    - Yes → Go to question 17
    - No → Go to question 19
17. Have you used the option to have GameBus automatically record your physical activities for you?
    - Yes → Go to question 19
    - No → Go to question 18
18. Why didn't you take advantage of the option to have GameBus automatically record activities for you?

Communication & Rewards

19. Via what communication channels were you informed about the Beweeg & Scoor program?
20. I find communication on the that the Beweeg & Scoor program clear.
    - Strongly agree
    - Agree
    - Neutral
    - Disagree
    - Strongly disagree
21. Did you have the opportunity to win prizes throughout the Beweeg & Scoor program?
    - Yes → Go to question 22
    - No → Go to question 24
22. What prizes could you have won?
23. Have you won one or more prizes?
    - Yes → Go to question 24
    - No → Go to question 25
24. What prizes did you win?

Personality traits

How much do you agree with each statement about you as you generally are now, not as you wish to be in the future?

25. I am the life of the party.
    - Strongly agree
    - Agree
    - Neutral
    - Disagree

- Strongly disagree

26. I sympathize with others’ feelings.
    - Strongly agree
    - Agree
    - Neutral
    - Disagree
    - Strongly disagree
27. I get chores done right away.
    - Strongly agree
    - Agree
    - Neutral
    - Disagree
    - Strongly disagree
28. I have frequent mood swings.
    - Strongly agree
    - Agree
    - Neutral
    - Disagree
    - Strongly disagree
29. I have a vivid imagination.
    - Strongly agree
    - Agree
    - Neutral
    - Disagree
    - Strongly disagree
30. I don’t talk a lot.
    - Strongly agree
    - Agree
    - Neutral
    - Disagree
    - Strongly disagree
31. I am not interested in other people’s problems.
    - Strongly agree
    - Agree
    - Neutral
    - Disagree
    - Strongly disagree
32. I often forget to put things back in their proper place.
    - Strongly agree
    - Agree
    - Neutral
    - Disagree
    - Strongly disagree
33. I am relaxed most of the time.
    - Strongly agree
    - Agree
    - Neutral

- Disagree
- Strongly disagree

34. I am not interested in abstract ideas.
    - Strongly agree
    - Agree
    - Neutral
    - Disagree
    - Strongly disagree
35. I talk to a lot of different people at parties.
    - Strongly agree
    - Agree
    - Neutral
    - Disagree
    - Strongly disagree
36. I feel others' emotions
    - Strongly agree
    - Agree
    - Neutral
    - Disagree
    - Strongly disagree
37. I like order.
    - Strongly agree
    - Agree
    - Neutral
    - Disagree
    - Strongly disagree
38. I get upset easily.
    - Strongly agree
    - Agree
    - Neutral
    - Disagree
    - Strongly disagree
39. I have difficulty understanding abstract ideas.
    - Strongly agree
    - Agree
    - Neutral
    - Disagree
    - Strongly disagree
40. I keep in the background.
    - Strongly agree
    - Agree
    - Neutral
    - Disagree
    - Strongly disagree
41. I am not really interested in others.
    - Strongly agree
    - Agree

- Neutral
- Disagree
- Strongly disagree

42. I make a mess of things.
    - Strongly agree
    - Agree
    - Neutral
    - Disagree
    - Strongly disagree
43. I seldom feel blue.
    - Strongly agree
    - Agree
    - Neutral
    - Disagree
    - Strongly disagree
44. I do not have a good imagination.
    - Strongly agree
    - Agree
    - Neutral
    - Disagree
    - Strongly disagree

Other feedback and future outlook

45. How likely are you to recommend the GameBus app to a colleague?
    - 01
    - 02
    - 03
    - 04
    - 05
    - 06
    - 07
    - 08
    - 09
    - 10
46. What experience (either positive and/or negative) with the Beweeg & Scoor program would you like to share?
47. May we contact you in response to your answers?
    - Yes
    - No
48. In what physical activity do you currently engage, and where?
49. What are your future physical activity goals?
50. What support may your employer offer to accommodate your physical activity goals?
