# Supplementary material for: Evaluating the Impact of Adaptive Personalized Goal Setting on Engagement Levels of Government Staff With a Gamified mHealth Tool: Results From a 2-Month Randomized Controlled Trial
Source: JMIR Mhealth Uhealth. 2022 Mar 31;10(3):e28801. doi: 10.2196/28801 (PMC9015741; doi:10.2196/28801)
Supplement: Multimedia Appendix 2 [file mhealth_v10i3e28801_app2.pdf]

Detailed output of statistical analysis

S1

$$dropouts \sim wave + treatment$$

Predictor	Estimate	Std. error	T-value	P-value
(intercept)	10.625	3.272	3.248	0.048
wave = first	16.000	4.138	3.866	0.031
wave = second	7.500	4.138	1.812	0.168
wave = third	0.000	4.138	0.000	1.000
treatment = control	-0.250	2.926	-0.085	0.937

S2

$$\ln(days\ online) \sim relwave + goal + (1 \mid user) + (1 \mid organization)$$

Random effect	Name	Variance	Std. dev.
user	(intercept)	0.477	0.690
organization	(intercept)	0.037	0.191
residual		0.326	0.571

Fixed effect	Estimate	Std. error	DF	T-value	P-value
(intercept)	0.999	0.170	9.503	5.873	< 0.001
rel. wave	-0.161	0.038	172.208	-4.227	< 0.0001
goal = maintain	0.778	0.221	95.003	3.519	< 0.001
goal = improve	0.486	0.171	101.351	2.839	0.005

S3

$$\sqrt{activities} \sim relwave + goal + (1 \mid user) + (1 \mid organization)$$

Random effect	Name	Variance	Std. dev.
user	(intercept)	3.577	1.891
organization	(intercept)	0.043	0.208
residual		1.320	1.149

Fixed effect	Estimate	Std. error	DF	T-value	P-value
(intercept)	2.132	0.365	9.503	6.433	< 0.001
relwave	-0.283	0.078	172.208	164.723	< 0.001
goal = maintain	1.239	0.569	95.003	98.714	0.032
goal = improve	1.805	0.437	101.351	103.449	< 0.0001

S4

$$longer\ walks \sim goal\ +\ (1\,|\,user)\ +\ (1\,|\,organization)$$

Random effect	Name	Variance	Std. dev.
user	(intercept)	2.208	1.486
organization	(intercept)	0.214	0.462
residual		0.026	0.161

Fixed effect	Estimate	Std. error	DF	T-value	P-value
(intercept)	0.066	0.312	11.517	0.213	0.835
goal = maintain	0.261	0.412	105.446	0.633	0.528
goal = improve	0.917	0.312	105.282	2.934	0.004

S5

$$sports\ sessions \sim treatment +\ goal\ +\ (1\,|\,user)\ +\ (1\,|\,organization)$$

Random effect	Name	Variance	Std. dev.
user	(intercept)	0.524	0.724
organization	(intercept)	0.007	0.081
residual		0.008	0.090

Fixed effect	Estimate	Std. error	DF	T-value	P-value
(intercept)	-0.050	0.147	24.562	-0.342	0.735
treatment = personalized	0.276	0.141	107.117	1.955	0.053
goal = maintain	0.405	0.200	103.560	2.020	0.046
goal = improve	0.318	0.154	103.512	2.063	0.042

S6

$$\sqrt{bike\ performance} \sim treatment$$

Predictor	Estimate	Std. error	T-value	P-value
(intercept)	1.682	0.070	23.970	< 0.0001
treatment = personalized	0.217	0.102	2.131	0.040

S7

$$sports\ performance \sim treatment\ \cdot goal$$

Predictor	Estimate	Std. error	T-value	P-value
(intercept)	3.500	0.486	7.208	< 0.0001
treatment = personalized	0.500	0.687	0.728	0.472
goal = maintain	-1.250	0.687	-1.820	0.039
goal = improve	0.250	0.561	0.446	0.039
treatment = personalized & goal = maintain	1.250	1.011	1.237	0.037
treatment = personalized & goal = improve	-0.978	0.798	-1.225	0.037

S8

$$perceived\ capability \sim number\ of\ waves\ online$$

Predictor	Estimate	Std. error	T-value	P-value
(intercept)	3.937	0.384	10.265	< 0.0001
number of waves online	-0.329	0.095	-3.452	0.001
