# Supplementary material for: Evaluating the Impact of Adaptive Personalized Goal Setting on Engagement Levels of Government Staff With a Gamified mHealth Tool: Results From a 2-Month Randomized Controlled Trial
Source: JMIR Mhealth Uhealth. 2022 Mar 31;10(3):e28801. doi: 10.2196/28801 (PMC9015741; doi:10.2196/28801)
Supplement: Multimedia Appendix 3 [file mhealth_v10i3e28801_app3.pdf]

Municipality	Treatment	Participants	Short walks	Longer walks	Short bike rides	Longer bike rides	Sports sessions	Other	Total
Brasschaat	Control	7	5	0	0	5	0	0	10
Brasschaat	Personalized	6	4	0	0	0	0	0	4
Essen	Control	20	325	115	66	32	15	6	559
Essen	Personalized	32	352	100	53	95	63	8	671
Kapellen	Control	14	2	3	10	6	3	0	24
Kapellen	Personalized	11	11	7	0	21	19	0	58
Kapellen	N.A.	1	0	0	0	0	0	0	0
Schoten	Control	2	0	0	3	0	5	0	8
Schoten	Personalized	1	0	0	0	0	0	0	0
Stabroek	Control	9	0	0	0	0	0	0	0
Stabroek	Personalized	14	0	0	0	0	0	0	0
Wuustwezel	Control	28	105	55	62	70	22	14	328
Wuustwezel	Personalized	19	213	100	5	55	22	4	399
Wuustwezel	N.A.	1	0	0	0	0	0	0	0
Sport Vlaanderen	Control	2	0	0	0	0	0	0	0
Sport Vlaanderen	Personalized	1	0	0	0	0	0	0	0
Sport Vlaanderen	N.A.	8	0	0	0	0	0	0	0
